# The Great Mimicker: Acute Obstructive Uropathy as a Rare Manifestation of Long-Standing Pseudomyxoma Peritonei

**DOI:** 10.7759/cureus.111122

**Published:** 2026-06-18

**Authors:** Paolo Romero Merino, Carmen R Pozo Morales, Ronald J Alvarez Guzman

**Affiliations:** 1 Family Medicine, Gerencia de Atención Primaria Valladolid Este, Valladolid, ESP; 2 Faculty of Medicine, Ricardo Palma University, Institute of Research in Biomedical Sciences, Lima, PER

**Keywords:** case report, conservative management, obstructive uropathy, peritoneal carcinomatosis, pseudomyxoma peritonei

## Abstract

Pseudomyxoma peritonei (PMP) is a rare clinical entity characterized by the accumulation of mucinous fluid within the peritoneal cavity. While often indolent, it can lead to severe mechanical complications. This case describes an elderly patient with advanced PMP presenting with acute obstructive uropathy, highlighting the complexity of managing frail oncologic patients. An 83-year-old female with a history of ovarian cancer and PMP (previously treated with cytoreductive surgery and HIPEC (hyperthermic intraperitoneal chemotherapy)) presented with a two-day history of right-sided flank pain, abdominal distention, and severe constipation. Laboratory results showed leukocytosis (13,790/µL) and elevated C-reactive protein (169 mg/L). A CT scan revealed progression of calcified peritoneal implants and a large epigastric mass causing extrinsic compression of the right ureter, leading to severe ureterohydronephrosis (7 cm). Despite the obstruction, renal function was preserved (creatinine 0.73 mg/dL). Due to her frailty and personal preferences, the patient declined invasive urinary diversion. She was managed conservatively, remained clinically stable, and was discharged with outpatient follow-up. PMP symptoms often mimic benign gastrointestinal issues, such as constipation. In elderly patients, the decision between aggressive intervention and conservative care is challenging. This case highlights the value of shared decision-making in advanced oncology; when renal function is stable, this model allows for a non-invasive approach that, beyond being a viable clinical alternative, stands as the best path to honor patient autonomy and optimize quality of life. Clinical vigilance is crucial in PMP, as nonspecific symptoms may mask serious obstructive complications. Individualized, patient-centered care is essential when balancing the risks of surgical intervention against conservative management in advanced oncology.

## Introduction

Abdominal and flank pain in elderly patients poses a significant diagnostic challenge because clinical manifestations are often nonspecific and may be influenced by multiple comorbidities, underlying malignancy, and atypical disease presentations. In this population, delayed recognition of uncommon intra-abdominal processes may lead to significant morbidity [1].

Pseudomyxoma peritonei (PMP) is a rare clinicopathologic condition characterized by the progressive intraperitoneal accumulation of mucinous ascites, most commonly arising from appendiceal mucinous neoplasms and less frequently associated with ovarian involvement or other mucin-producing tumors [2]. Although PMP often follows an indolent course, it may behave in a locally aggressive manner, causing progressive abdominal distension, pain, bowel dysfunction, and compression of adjacent intra-abdominal or retroperitoneal structures [3].

The standard treatment for selected patients consists of cytoreductive surgery combined with hyperthermic intraperitoneal chemotherapy (HIPEC), an approach associated with improved long-term outcomes [4]. However, this strategy may not be feasible in elderly or frail patients, in whom disease burden, comorbidities, and functional status frequently necessitate a more individualized approach. Because symptoms such as abdominal discomfort, constipation, distension, or poorly localized pain may be attributed to more common gastrointestinal or urinary conditions, the diagnosis of PMP-related complications can be particularly challenging, especially in patients with complex oncologic histories [5].

Recognition of atypical presentations is therefore essential to guide appropriate imaging, avoid diagnostic delay, and inform management decisions. We present the case of an elderly woman with a history of ovarian malignancy and persistent PMP, with an unclear primary origin, who developed right-sided flank pain and abdominal distension, illustrating the diagnostic complexity of atypical symptom presentation in patients with advanced peritoneal disease.

## Case presentation

An 83-year-old woman presented to the emergency department with a two-day history of right-sided flank pain associated with lower abdominal discomfort and nausea. The pain was described as colicky, radiating to the back, and accompanied by progressive abdominal distension. She denied fever, chills, or vomiting. She reported urinary frequency without dysuria and a long-standing history of chronic constipation.

Given her advanced age, her baseline functional status was evaluated, demonstrating an Eastern Cooperative Oncology Group (ECOG) performance status of 2 (capable of all self-care but unable to carry out any work activities) and a score of 4 on the Clinical Frailty Scale (CFS), indicating moderate vulnerability. Her activities of daily living (ADLs) were partially preserved; she ambulated with minimal assistance, and no drastic recent weight loss was noted during her last nutritional evaluation.

The patient’s detailed oncological history revealed a complex, long-standing course. In 2007, she underwent initial surgery for peritoneal carcinomatosis secondary to disseminated ovarian cancer, which included an omentectomy, hysterectomy, bilateral adnexectomy, and appendectomy. A subsequent re-evaluation CT scan revealed residual disease from PMP. Although the patient initially declined cytoreduction with intraperitoneal chemotherapy, she eventually accepted the intervention following clinical and radiological progression. She underwent a Sugarbaker-type cytoreductive surgery with a peritonectomy combined with HIPEC. Years later, she required a second cytoreductive surgery due to the progression of peritoneal implants. Finally, during her last in-person consultation with the Oncology service in January 2024, the case was thoroughly discussed with the patient. As she was an elderly, malnourished patient with a very slow-growing tumor and an expected low response to systemic chemotherapy, it was determined that the potential toxicity of a platinum-based regimen outweighed the clinical benefit. At that point, a strategy of watchful waiting and conservative management was established, reserving surgical interventions exclusively for acute obstructive symptoms.

On arrival, the patient was hemodynamically stable, with a blood pressure of 167/61 mmHg, heart rate of 76 beats per minute, oxygen saturation of 96% on room air, and a temperature of 37.4°C. Physical examination revealed a well-appearing, oriented patient in no acute distress. Cardiopulmonary examination was unremarkable. Abdominal examination demonstrated preserved bowel sounds, diffuse tenderness, and palpable fecal masses consistent with severe constipation. There were no signs of peritoneal irritation, and no clinically evident bladder distension was noted.

Laboratory studies revealed leukocytosis of 13,790/µL with neutrophil predominance (88.4%). Hemoglobin was 10.1 g/dL, and platelets were elevated at 490,000/µL. Serum sodium was decreased at 130 mmol/L, while renal function remained preserved with a creatinine of 0.73 mg/dL. Inflammatory markers were significantly elevated, with a C-reactive protein of 169 mg/L. Urinalysis showed a pH of 6.5 and low density (1.006). The automated strip was positive for leukocytes (++) and showed traces of proteins. Microscopic sediment examination confirmed 20-40 leukocytes per field and 5-10 erythrocytes per field, indicating an active inflammatory process in the urinary tract. Urine culture results were pending at the time of evaluation (Table 1).

**Table 1:** Admission Laboratory Results Asterisks (*) indicate values outside the laboratory’s reference range. The initial urine culture was reported as contaminated; a repeat culture was not performed due to the patient's favorable clinical response and stabilization of inflammatory markers under conservative management. Abbreviations: ALT/GPT: Alanine aminotransferase; CKD-EPI: Chronic Kidney Disease Epidemiology Collaboration; RBC: Red blood cells.

Test	Result	Units	Reference Range
Blood Biochemistry			
Sodium*	130	mmol/L	136–145
Potassium	3.8	mmol/L	3.5–5.3
Chloride	96	mmol/L	90–110
Calcium	8.97	mg/dL	8.6–10.4
Glucose	110	mg/dL	74–110
Urea	27	mg/dL	19–47
Creatinine	0.73	mg/dL	0.5–1.1
CKD-EPI	76	mL/min/1.73 m²	
ALT/GPT	16	U/L	10–35
Total Bilirubin	0.23	mg/dL	0–1.2
Alkaline Phosphatase*	243	U/L	35–104
Lipase	20	U/L	13–60
Phosphate	2.9	mg/dL	2.5–5
C-Reactive Protein*	169	mg/L	0–5
Procalcitonin	0.16	ng/mL	0–0.5
Automated Urinalysis Dipstick			
Specific Gravity	1.006		1.005–1.031
pH	6.5		5–8
Leukocytes*	Positive ++	-	-
Erythrocytes (RBC)	Negative	-	-
Nitrites	Negative	-	-
Glucose	Negative	-	-
Proteins	Traces	-	-
Bilirubin	Negative	-	-
Ketones	Negative	-	-
Urobilinogen	Negative	-	-
Automated Urine Sediment			
Leukocytes*	20–40	/ Field	0–5 / Field
Erythrocytes*	5–10	/ Field	0–3 / Field
Squamous epithelial cells	Few	-	-
Bacteria	Few	-	-
Hematology			
White Blood Cell Count			
Leukocytes*	13.79	×10^3^/µL	4–10
Neutrophils %*	88.4	%	42–76
Lymphocytes %*	3.8	%	20–51
Monocytes %	7.5	%	2–12
Eosinophils %	0.1	%	0–8
Basophils %	0.2	%	0–2
Red Blood Cell Count			
RBC (Red Blood Cells)	3.5	×10^6^/µL	4.2–5.2
Hemoglobin*	10.1	g/dL	12–16
Hematocrit*	31.4	%	36–46
Platelets*	490	×10^3^/µL	150–400

Given the patient's symptoms and complex oncological history, an emergency contrast-enhanced abdominal CT scan was performed. The images demonstrated a large, multiloculated peritoneal mass in the epigastric and mesogastric regions, consistent with her known history of PMP, with a progression in the number and size of calcified peritoneal implants. Other findings included subcapsular hepatic implants in segment VI and multiple calcified peritoneal deposits throughout the abdominal cavity, including the umbilical region. No pneumoperitoneum, intra-abdominal fluid collections, or bowel obstruction was identified.

The emergency contrast-enhanced abdominal CT scan and renal ultrasound confirmed a grade IV hydronephrosis of the right kidney, characterized by a massive dilatation of the renal pelvis measuring up to 7 cm in its greatest diameter, along with severe caliceal ballooning. Despite this profound structural dilation, the renal cortical thickness remained remarkably preserved, showing no signs of parenchymal atrophy or cortical thinning. This advanced obstructive uropathy was secondary to a critical focal narrowing of the right ureter at its middle third, where the ureteral lumen was concentrically compressed to a filiform caliber due to the extrinsic mass effect and encasement exerted by the progressive, partially calcified mucinous peritoneal implants (Figures 1, 2).

**Figure 1:**
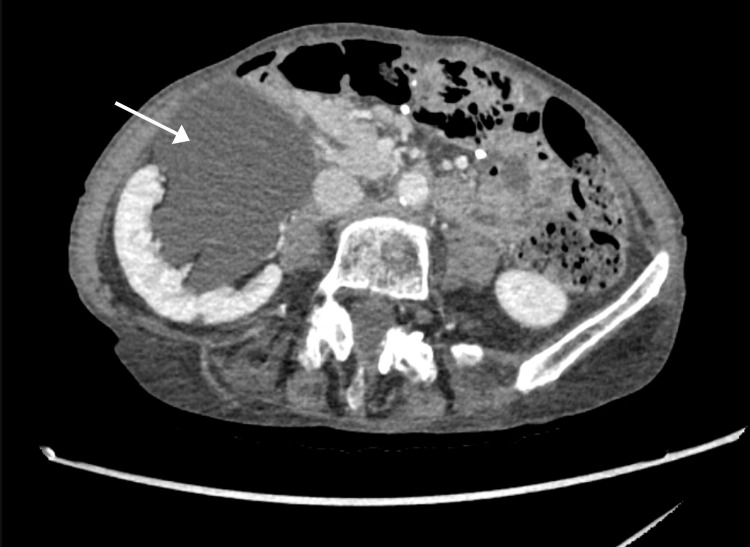
Abdominal CT (axial): contrast-enhanced abdominal CT scan showing severe right-sided hydronephrosis (white arrow). There is marked dilation of the renal pelvis with preserved renal cortical thickness, resulting from extrinsic compression by mucinous peritoneal implants in the setting of pseudomyxoma peritonei

**Figure 2:**
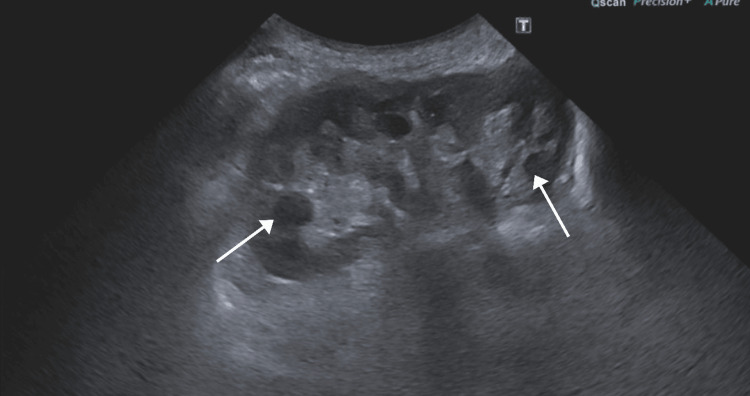
Right renal ultrasound: marked dilation of the collecting system (grade IV hydronephrosis), characterized by dilated calyces (white arrows), consistent with extrinsic urinary tract obstruction

When comparing the emergency abdominopelvic CT scan with the previous tomographic study available from December 2023, it was evident that the large multiseptated peritoneal mass in the epigastrium-mesogastrium and the subcapsular hepatic implants in segment VI were already present beforehand. However, the current study demonstrated a clear progression in the number and size of the calcified peritoneal implants, which increased by approximately 1 cm over the last year, leading to the extrinsic ureteral compression and subsequent grade IV hydronephrosis.

Regarding the marked urinary bladder distension observed on imaging, an organic bladder outlet obstruction was clinically ruled out through physical examination (absence of a painful palpable bladder) and post-void ultrasound. This finding was interpreted as a secondary manifestation of extrinsic extraluminal compression on the bladder dome caused by the extensive peritoneal disease affecting all quadrants, compounded by age-related functional changes. Consequently, urinary retention was not considered a primary or aggravating component of the right hydronephrosis. Based on these findings, a diagnosis of malignant right obstructive uropathy secondary to extrinsic ureteral compression by peritoneal carcinomatosis in the context of persistent PMP was established.

The patient was admitted to the urology service. The placement of a double-J ureteral stent was recommended to relieve the obstruction; however, after consulting with her family, she declined invasive urinary diversion due to her underlying oncological condition and personal preferences. In view of her preserved renal function (creatinine 0.73 mg/dL) and a normal contralateral kidney, a strictly conservative, medical, and symptomatic management approach was chosen, accompanied by rigorous clinical and biochemical monitoring. During hospitalization, the patient remained afebrile and hemodynamically stable. Consequently, despite the initial urinary sediment findings (leukocytosis and protein traces), empirical antibiotic therapy was withheld - a decision supported by a remarkably low procalcitonin (PCT) value of 0.16 ng/mL and an initial urine culture reported as contaminated. Pain in both flanks and the hypogastrium was optimally managed with conventional, non-nephrotoxic analgesia.

Furthermore, urine output remained within normal limits, and follow-up laboratory analyses demonstrated a clear improvement, highlighted by the resolution of leukocytosis (9,010/µL), stable renal function (creatinine 0.64 mg/dL), and a generalized decrease in inflammatory markers. Given this favorable clinical course and the absence of complications, the patient was discharged with outpatient follow-up indications and precise instructions for close clinical monitoring.

## Discussion

PMP is an uncommon clinicopathological entity characterized by progressive intraperitoneal accumulation of mucinous ascites and dissemination of mucin-producing epithelial implants, most frequently originating from appendiceal neoplasms, although ovarian involvement may coexist or represent secondary implants. Its estimated incidence is low, which contributes to limited clinician familiarity and frequent diagnostic delays [6,7]. The present case illustrates several classical and atypical features of PMP, including chronic indolent progression, recurrent peritoneal disease despite prior cytoreductive treatment, and the late development of urinary tract obstruction.

The natural history of PMP is usually slow but relentless, with progressive redistribution of mucin and tumor cells throughout dependent areas of the peritoneal cavity. As a result, patients often present with nonspecific manifestations such as increasing abdominal girth, vague abdominal pain, nausea, early satiety, altered bowel habits, or incidental radiologic findings [7,8]. In elderly patients, these symptoms may be mistakenly attributed to more common benign disorders, particularly constipation or functional gastrointestinal disease. In our patient, long-standing constipation and palpable fecal loading initially represented a plausible explanation for abdominal discomfort, potentially obscuring a more significant underlying complication.

Given this scenario, flank pain in an elderly patient necessitates a meticulous differential diagnosis of prevalent urological conditions. Unlike acute nephrolithiasis, where pain is classically sudden, excruciating, and strongly associated with frank gross or microscopic hematuria [9], our patient presented with an intermittent subacute pain exacerbated by abdominal distension. Furthermore, the absence of high fever, exquisite costovertebral angle tenderness, and low procalcitonin levels ruled out acute pyelonephritis, despite the initial leukocytosis. Lastly, acute urinary retention typically presents with severe suprapubic pain, urinary incapacitation, and acute cognitive agitation in older adults [10]; features that were absent in this case, where the bladder distension seen on imaging represented an incidental, non-obstructive finding.

From a pathophysiological perspective, extrinsic ureteral compression in PMP does not result from direct tumor invasion of the urinary mucosa, but rather from a unique mechanical and cellular mechanism. Floating mucinous epithelial cells accumulate in the dependent areas of the retroperitoneum and pelvis due to the "redistribution" phenomenon. Once implanted, these cells continuously secrete large volumes of dense, gelatinous mucin. Over time, this mucinous material undergoes a stromal inflammatory response that induces fibrosis and peripheral calcification of the implants, transforming them into dense, semi-solid masses. When located in the retroperitoneal space, these masses encircle and concentrically encase the ureter. The external hydrostatic pressure exerted by the indolent growth of these calcified implants eventually exceeds the intraluminal ureteral pressure, progressively collapsing it and leading to severe obstructive uropathy without requiring luminal infiltration [8,11]. This mechanism explains why patients can remain clinically stable for prolonged periods and then present with abrupt symptoms when a critical structure becomes obstructed.

A particularly relevant aspect of this case was the presence of severe right-sided hydronephrosis secondary to extrinsic ureteral compression. Urinary tract involvement in PMP is considered uncommon when compared with intestinal obstruction or nutritional compromise, but it may occur in advanced or recurrent disease, especially in patients with pelvic implants, prior surgery, fibrosis, or extensive locoregional progression [11,12]. The patient’s colicky flank pain radiating to the back is physiologically consistent with acute urinary tract obstruction and should prompt consideration of obstructive uropathy in similar clinical contexts.

Notably, renal function remained preserved despite marked dilation of the collecting system. This finding can be explained by unilateral obstruction with contralateral kidney compensation, as well as a possible subacute evolution that allows physiological adaptation. Nevertheless, persistent hydronephrosis carries the risk of progressive nephron loss, infection, pain recurrence, and eventual renal impairment if left untreated [12,13]. Therefore, timely recognition remains clinically important even when serum creatinine is initially normal.

Cross-sectional imaging was decisive in this case. Contrast-enhanced computed tomography simultaneously demonstrated progression of peritoneal implants, excluded bowel obstruction or perforation, and identified the urinary tract complication. CT remains the imaging modality of choice in many PMP patients because it allows assessment of mucin distribution, calcified implants, visceral scalloping, hydronephrosis, and treatment response over time [13,14]. Imaging is particularly valuable when symptoms are nonspecific or multifactorial, as occurred in this patient with overlapping constipation, abdominal pain, and flank pain.

Regarding treatment, complete cytoreductive surgery combined with hyperthermic intraperitoneal chemotherapy (CRS-HIPEC) is currently regarded as the standard potentially disease-controlling strategy in selected patients with resectable disease and adequate physiological reserve [14,15]. Long-term survival outcomes are significantly better when complete cytoreduction is achieved, particularly in low-grade histology. However, treatment-related morbidity is not negligible, and outcomes depend heavily on patient selection, tumor biology, disease extent, and institutional expertise.

In the present case, several factors argued against additional aggressive intervention: advanced age, frailty, prior extensive abdominal surgeries, persistent disease despite previous CRS-HIPEC, and relatively indolent oncologic behavior. These circumstances are consistent with literature emphasizing that management of recurrent or persistent PMP should be individualized rather than automatically surgical [15,16]. In selected elderly patients, symptom-focused conservative management may represent the most proportionate strategy.

Ureteral stenting was appropriately proposed as a less invasive decompressive option. However, the patient declined urinary diversion after discussion with her family and treating team. Respect for patient autonomy is central to modern oncologic care, particularly when interventions may offer anatomical improvement without clear survival benefit or may negatively affect quality of life [16,17]. Shared decision-making is especially relevant in geriatric oncology, where goals of care frequently prioritize comfort, independence, and minimization of procedural burden.

Another instructive element of this report is the discordance between elevated inflammatory markers and low procalcitonin. In patients with advanced malignancy, C-reactive protein may rise due to tumor-associated inflammation, tissue stress, or nonbacterial inflammatory states, whereas low procalcitonin can reduce suspicion of systemic bacterial infection in the appropriate context [17]. This distinction may help avoid premature attribution of symptoms solely to infection when structural complications are present.

This case also highlights the need for broad differential diagnosis when patients with known PMP present with new pain syndromes. Flank pain in such patients should not be assumed to be musculoskeletal or benign without evaluating nephrolithiasis, pyelonephritis, urinary retention, or malignant ureteral compression. Similarly, abdominal distension may reflect constipation, tumor progression, ascites, bowel obstruction, or combined processes occurring simultaneously [18].

## Conclusions

This case highlights the diagnostic complexity of abdominal and flank pain in elderly patients with PMP, in whom nonspecific symptoms may mask clinically significant complications. In our patient, right-sided flank pain was ultimately attributable to obstructive uropathy caused by extrinsic ureteral compression from extensive peritoneal disease, an uncommon but important manifestation. From a clinical perspective, this report emphasizes the need to consider both disease-related complications and coexisting benign conditions, such as severe constipation, when evaluating patients with complex oncologic histories. Careful imaging assessment is essential to avoid misattribution of symptoms and delayed diagnosis.

Furthermore, this case underscores the importance of individualized, patient-centered management. While cytoreductive surgery and HIPEC remain the standard of care for selected patients, conservative management may be appropriate in elderly or frail individuals when the risks of intervention outweigh potential benefits. Overall, recognizing atypical presentations of PMP and tailoring management to the patient’s clinical context are critical to optimizing outcomes and preserving quality of life.
